# Safety, tolerability, and pharmacokinetics of Aurora kinase B inhibitor AZD2811: a phase 1 dose-finding study in patients with advanced solid tumours

**DOI:** 10.1038/s41416-023-02185-2

**Published:** 2023-03-04

**Authors:** Melissa L. Johnson, Judy S. Wang, Gerald Falchook, Carol Greenlees, Suzanne Jones, Donald Strickland, Giulia Fabbri, Caroline Kennedy, J. Elizabeth Pease, Liz Sainsbury, Alexander MacDonald, Stein Schalkwijk, Philip Szekeres, Jan Cosaert, Howard Burris

**Affiliations:** 1 Sarah Cannon Research Institute, Nashville, TN USA; 2 Tennessee Oncology, Nashville, TN USA; 3 Florida Cancer Specialists/Sarah Cannon Research Institute, Sarasota, FL USA; 4 Sarah Cannon Research Institute at HealthONE, Denver, CO USA; 5 AstraZeneca, Waltham, MA USA; 6 AstraZeneca, Cambridge, UK; 7 Present Address: Avacta Life Sciences, London, UK; 8 Present Address: GlaxoSmithKline, London, UK

**Keywords:** Targeted therapies, Targeted therapies, Targeted therapies

## Abstract

**Background:**

AZD2811 is a potent, selective Aurora kinase B inhibitor. We report the dose-escalation phase of a first-in-human study assessing nanoparticle-encapsulated AZD2811 in advanced solid tumours.

**Methods:**

AZD2811 was administered in 12 dose-escalation cohorts (2-h intravenous infusion; 15‒600 mg; 21-/28-day cycles) with granulocyte colony-stimulating factor (G-CSF) at higher doses. The primary objective was determining safety and maximum tolerated/recommended phase 2 dose (RP2D).

**Results:**

Fifty-one patients received AZD2811. Drug exposure was sustained for several days post-dose. The most common AZD2811-related adverse events (AEs) were fatigue (27.3%) at ≤200 mg/cycle and neutropenia (37.9%) at ≥400 mg/cycle. Five patients had dose-limiting toxicities: grade (G)4 decreased neutrophil count (*n* = 1, 200 mg; Days 1, 4; 28-day cycle); G4 decreased neutrophil count and G3 stomatitis (*n* = 1 each, both 400 mg; Day 1; 21-day cycle); G3 febrile neutropenia and G3 fatigue (*n* = 1 each, both 600 mg; Day 1; 21-day cycle +G-CSF). RP2D was 500 mg; Day 1; 21-day cycle with G-CSF on Day 8. Neutropenia/neutrophil count decrease were on-target AEs. Best overall responses were partial response (*n* = 1, 2.0%) and stable disease (*n* = 23, 45.1%).

**Conclusions:**

At RP2D, AZD2811 was tolerable with G-CSF support. Neutropenia was a pharmacodynamic biomarker.

**Clinical trial registration:**

NCT02579226.

## Introduction

The Aurora kinases are a family of serine/threonine kinases that play a critical role in the regulation of mitosis and chromosome alignment, accurate chromosomal segregation, and cytokinesis [1]. Aurora kinase B (AURKB) is responsible for phosphorylation of Ser10 of histone H3, which is required for correct chromosome condensation during mitosis [2]. AURKB is frequently overexpressed in various solid tumours and haematological malignancies [3–9], leading to chromosomal instability [10, 11], and is associated with poor prognosis [11–14]. Several AURKB inhibitors have been investigated in phase 1–2 clinical trials, with success in some solid tumours, especially those with rapid cell turnover and dependency on efficient mitoses, such as small cell lung cancer (SCLC) [15–21].

AZD2811, formerly designated AZD1152 hydroxyquinazoline pyrazole anilide (AZD1152 hQPA), is an intravenously administered, potent and selective inhibitor of AURKB activity [22]. Inhibition of AURKB induces chromosome misalignments during mitosis and failed cytokinesis, leading to polyploidy and eventually to cell death [5]. Barasertib (AZD1152) is a prodrug of AZD2811 that converts rapidly to the active drug in plasma. In previous research, barasertib showed promising efficacy in the treatment of acute myeloid leukaemia (AML), but had to be administered as a continuous 7-day intravenous (IV) infusion [17]. In patients with advanced solid tumours, the use of barasertib was limited by frequent bone marrow toxicities and poor clinical response [18]. The requirement for continuous IV infusion and the toxicity profile of the prodrug limited its utility, leading to the development of an alternative formulation in which the active drug is incorporated into a nanoparticle carrier, allowing extended release of the active payload [23]. This formulation provides an extended exposure profile when administered as a 2- to 4-h infusion similar to that of the barasertib 7-day infusion. In animal models of SCLC and diffuse large B-cell lymphoma (DLBCL), AZD2811 showed greater efficacy and less toxicity at half the dose intensity of barasertib [23, 24].

This first-in-human, phase 1 study was conducted to determine the maximum tolerated dose (MTD), recommended phase 2 dose (RP2D), dosing schedule, safety, tolerability, and pharmacokinetics (PK) of AZD2811 as second-line or later therapy in patients with advanced solid tumours (NCT02579226; also called REFMAL 390) with disease progression or intolerance to standard therapies, or for whom no standard of care existed. The study consisted of Part A (dose escalation) and Part B (dose expansion in patients with SCLC). Here we report the results of Part A.

## Methods

### Trial design

This multicentre, open-label study was conducted in the USA. The dose-escalation schema is shown in Supplementary Fig. 1.

### Participants

The study enrolled patients aged ≥18 years, with histological or cytological confirmation of a solid tumour. They could have received ≤3 prior chemotherapy regimens in the metastatic setting. Other key inclusion criteria included Eastern Cooperative Oncology Group performance status (ECOG PS) 0–1, measurable or non-measurable (but evaluable in non-target lesions) disease according to Response Evaluation Criteria in Solid Tumours version 1.1 (RECIST1.1), adequate organ function, and predicted life expectancy ≥12 weeks.

Patients must not have received radiotherapy, immunotherapy, chemotherapy or investigational drugs within 21 days or 5 half-lives (with a minimum of 21 days) of enrolment (screening), and could have no unresolved National Cancer Institute (NCI) Common Terminology Criteria for Adverse Events (CTCAE) v4.03 treatment-related adverse event (TRAE) of grade >1, with the exception of alopecia. Radiotherapy or surgery for brain metastases was allowed if therapy was completed ≥3 weeks previously, neurologic symptoms were mild, and there was no evidence of central nervous system disease progression or requirement for chronic corticosteroid therapy. Other key exclusion criteria included previous treatment with alisertib, grade ≥2 diarrhoea, major surgery ≤21 days or minor surgery ≤7 days from starting study drug, and pregnancy, lactation, or breastfeeding.

### Interventions

AZD2811 was administered as an IV infusion over 2 h. The study included 12 dose-escalation cohorts. In Cohorts 1–6, dosing took place on Days 1 and 4 of a 28-day cycle, at 15 mg, 25 mg, 38 mg, 51 mg, 100 mg, and 200 mg. In Cohort 1, the starting dose was to be administered to a single patient on Days 1 and 4. If this patient had a dose-limiting toxicity (DLT) or grade ≥2 adverse event (AE), more patients could be added to Cohort 1, up to a maximum of 6. Starting with Cohort 2, at least 3 evaluable patients were enrolled at each dose level (3 + 3 design) and were evaluated for 1 cycle before escalation to the next dose level. If one patient experienced a DLT, an additional 3 patients were treated with the same dose. Therefore, a maximum of 6 evaluable patients could be enrolled per dose level.

In Cohort 7, the Day 4 dose was removed from the schedule, and patients received AZD2811 200 mg on Day 1 of a 28-day cycle. Granulocyte colony-stimulating factor (G-CSF; filgrastim [short-acting G-CSF] or pegfilgrastim [long-acting G-CSF]) as primary prophylaxis for neutropenia was optional in Cohorts 7–9. In Cohorts 8–12, the cycle length was reduced, so dosing occurred on Day 1 of a 21-day cycle, at doses of 200–600 mg. Patients in Cohorts 10–12 received mandatory G-CSF on Day 8 after dosing in Cycle 1 as primary prophylaxis for neutropenia. PEGylated G-CSF (e.g. pegfilgrastim) could not be given later than Day 8 (in a 21-day cycle) due to its long half-life. G-CSF had to be discontinued at least 48 h prior to the next dose of AZD2811.

Patients could continue to receive AZD2811 as long as they continued to show clinical benefit, as judged by the investigator, and did not meet discontinuation criteria. Treatment could be discontinued due to patient decision, investigator decision, AEs, severe noncompliance with the protocol, confirmed disease progression, or incorrect initiation of the investigational drug.

A DLT was defined as any of the following toxicities that occurred during the DLT evaluation period (the first 28 days [if a 28-day cycle] or the first 21 days [if a 21-day cycle] from the start of AZD2811), unless unequivocally due to underlying malignancy or an extraneous cause: grade 4 neutropenia for >7 days or febrile neutropenia; grade 4 thrombocytopenia or grade 3 thrombocytopenia associated with bleeding; concurrent grade ≥3 elevation of total bilirubin, alanine aminotransferase (ALT), aspartate aminotransferase (AST) or alkaline phosphatase (ALP) lasting >48 h, or any change in liver function test results consistent with Hy’s Law; any grade ≥3 non-haematological AE (except grade 3 nausea, vomiting, stomatitis and/or diarrhoea that was controlled within 4 days with standard supportive care, or grade 3 elevations in ALT/AST that return to meet initial eligibility criteria within 7 days of study drug interruption); inability to receive all doses in Cycle 1 due to treatment-related toxicity; or grade ≥2 non-haematological toxicity at any time during treatment that was deemed to be dose-limiting by the investigator and the medical monitor. The MTD/RP2D was evaluated in patients who received all AZD2811 doses in Cycle 1 and completed all safety evaluations, or had a DLT in Cycle 1.

AEs were assessed throughout the treatment and follow-up periods. Patients were followed until all treatment-related toxicity resolved, or for at least 30 days post-study drug discontinuation. AEs were graded according to CTCAE v4.03. Venous blood samples (4 mL) for measurement of AZD2811 concentrations were collected at Cycle 1 Days 1–6, 8, 15, 16, and 22 and Cycle 2 Day 1 in Cohorts 1–6; Cycle 1 Days 1, 4, 8, 12, and 22 in Cohort 7; Cycle 1 Days 1, 4, 8, 12, and 15 and Cycle 2 Day 1 in Cohorts 8–12. Tumour imaging assessments using computed tomography or magnetic resonance imaging of the chest and abdomen/pelvis were performed at screening (≤28 days prior to initiation of treatment), and then every 2 cycles.

### Objectives and endpoints

The primary objective was to evaluate the safety profile and determine the MTD/RP2D and schedule of AZD2811 by assessing the incidence of DLTs, AEs, and abnormal laboratory test results. Secondary objectives were evaluation of PK parameters and preliminary assessment of antitumour activity, including objective response rate (ORR [complete response + partial response (CR + PR)]) based on RECIST v1.1, and incidence of best overall response, stable disease (SD) and progressive disease (PD).

### Statistical methods

The safety analysis set comprised all patients who received at least one dose of AZD2811. The PK analysis set comprised all patients who received at least one dose of AZD2811 with at least one reportable concentration. The evaluable-for-efficacy analysis set comprised all patients who received at least one dose of AZD2811 and had a baseline tumour assessment. There was no formal statistical analysis of safety and efficacy data. The numbers of patients experiencing each AE were summarised by Medical Dictionary for Regulatory Activities (MedDRA) version 22.1 system organ class, MedDRA preferred term, and CTCAE grade. The ORR and best overall response based on RECIST 1.1 were summarised. AZD2811 blood concentrations and derived PK parameters were summarised by dose regimen. Summary statistics were calculated for area under the curve (AUC), time of maximum concentration (*t*_max_), and maximum concentration after a single dose (*C*_max_).

## Results

### Demographics and baseline characteristics

Patients were enrolled from 16 November 2015 to 11 October 2018. At the time of database lock on 24 July 2020, 51 patients had been recruited into the dose-escalation phase and had received at least one dose of AZD2811: 24 patients in Cohorts 1–6 (AZD2811 administered on Days 1 and 4 of each cycle) and 27 patients in Cohorts 7–12 (AZD2811 administered on Day 1 of each cycle). Most cohorts consisted of 3 patients except for Cohorts 6 (9 patients), 7 (4 patients), 9 (8 patients), and 12 (6 patients); additional patients were recruited to ensure a sufficient number of evaluable patients for the DLT assessment (Supplementary Fig. 1 and Supplementary Table 1). Progressive disease was the main reason for treatment discontinuation (83.3% and 81.5% across Cohorts 1–6 and 7–12, respectively).

Patient demographics and disease characteristics are shown in Table 1. Median age was 61 years (range 38–81), 43.1% of patients were male, and the most common primary tumour types were lung (29.4%; comprising non-small-cell lung cancer (NSCLC) 13.7%, SCLC 13.7%, mesothelioma 2.0%), breast (19.6%), and prostate (11.8%). Patients were heavily pretreated: the median number of regimens of previous systemic therapy (chemotherapy, immunotherapy, hormonal therapy, targeted therapy) or radiotherapy for metastatic disease was 3 (range 1–9) across all cohorts.

**Table 1:** Patient demographics and disease characteristics.

	Total (*N* = 51)
Median age (range), years	61 (38–81)
Sex, *n* (%)	
Male	22 (43.1)
Female	29 (56.9)
Race, *n* (%)	
White	45 (88.2)
Black or African American	3 (5.9)
Other	1 (2.0)
Missing	2 (3.9)
ECOG PS, *n* (%)	
0	15 (29.4)
1	35 (68.6)
2	1 (2.0)^a^
Number of prior systemic therapies	
Median (range)	3 (1–9)
1 line, *n* (%)	7 (13.7)
2–3 lines, *n* (%)	25 (49.0)
>3 lines, *n* (%)	19 (37.3)
Best overall response to most recent prior regimen, *n* (%)	
CR	1 (2.0)
PR	6 (11.8)
Non-CR/non-PD	2 (3.9)
SD	16 (31.4)
PD	18 (35.3)
NE	1 (2.0)
NA	6 (11.8)
Missing	1 (2.0)
Primary diagnosis, *n* (%)	
Breast	10 (19.6)
Colon	3 (5.9)
Lung	15 (29.4)
NSCLC	7 (13.7)
SCLC	7 (13.7)
Mesothelioma	1 (2.0)
Ovary	2 (3.9)
Prostate	6 (11.8)
Other	15 (29.4)

*CR* complete response, *ECOG PS* Eastern Cooperative Oncology Group performance status, *NA* not available, *NE* not evaluable, *PD* progressive disease, *PR* partial response, *SD* stable disease.

^a^Patient enrolled in error.

### Study drug exposure

The median number of cycles was 4.0 in Cohorts 1–6 and 2.0 in Cohorts 7–12. The median treatment duration was 109 days (range 29–480) and 43 days (range 12–561), respectively. Notably, 1 patient in Cohort 8 received ≥24 cycles, 1 patient in Cohort 7 received ≥20 cycles, 1 patient in Cohort 6 received ≥17 cycles of treatment, 1 patient in Cohort 5 received ≥10 cycles, and 2 patients in Cohort 10 received ≥6 cycles of treatment.

### DLTs and determination of RP2D

All DLTs are shown in Table 2 and Supplementary Table 1. Among patients who received escalating doses of AZD2811 (15–200 mg) on Days 1 and 4 every 28 days in Cohorts 1–6, there were no DLTs in the first five cohorts (15–100 mg). In Cohort 6 (200 mg), 1 patient experienced a DLT of grade 4 decreased neutrophil count, necessitating the expansion of the cohort to 6 evaluable patients. From Cohort 7 onwards, AZD2811 was administered only on Day 1 of each cycle, and optional G-CSF on Day 8 was introduced. From Cohort 8 onwards, the cycle length was reduced from 28 to 21 days; Cohorts 7 and 8 (200 mg on Day 1 every 28 days and 200 mg on Day 1 every 21 days, respectively) were assessed concurrently, and no DLTs were reported in either cohort. In Cohort 9 (400 mg AZD2811 on Day 1 every 21 days), DLTs were reported in 2 of 6 evaluable patients (grade 3 stomatitis in 1 patient and grade 4 decreased neutrophil count in 1 patient, requiring treatment with G-CSF). The Safety Review Committee allowed enrolment in further cohorts at doses ≥400 mg only if G-CSF prophylaxis was mandatory; this applied from Cohort 10 onwards. Patients in Cohort 10 therefore received the same dose as Cohort 9, but with added G-CSF support on Day 8; no DLTs were reported. In Cohort 11 (600 mg on Day 1 every 21 days + G-CSF on Day 8), 2 out of 3 evaluable patients experienced DLTs (grade 3 febrile neutropenia in 1 patient and grade 3 fatigue in 1 patient). Consequently, in Cohort 12 the AZD2811 dose was reduced to 500 mg every 21 days, with mandatory G-CSF on Day 8, and there were no DLTs among the 6 evaluable patients; this dose was therefore determined to be the MTD/RP2D.

**Table 2:** All DLTs, SAEs, and AEs leading to discontinuation (safety analysis set).

	Days 1 and 4, 28-day cycles						Day 1, 28-day cycles	Day 1, 21-day cycles				
							Optional G-CSF			Mandatory G-CSF		
	Cohort 1: AZD2811 (15 mg) (*N* = 3)	Cohort 2: AZD2811 (25 mg) (*N* = 3)	Cohort 3: AZD2811 (38 mg) (*N* = 3)	Cohort 4: AZD2811 (51 mg) (*N* = 3)	Cohort 5: AZD2811 (100 mg) (*N* = 3)	Cohort 6: AZD2811 (200 mg) (*N* = 9)	Cohort 7: AZD2811 (200 mg) (*N* = 4)	Cohort 8: AZD2811 (200 mg) (*N* = 3)	Cohort 9: AZD2811 (400 mg) (*N* = 8)	Cohort 10: AZD2811 (400 mg) (*N* = 3)	Cohort 11: AZD2811 (600 mg) (*N* = 3)	Cohort 12: AZD2811 (500 mg) (*N* = 6)
*DLTs, n (%)*												
Total patients	0	0	0	0	0	1 (11.1)	0	0	2 (25.0)	0	2 (66.7)	0
Neutrophil count decreased	0	0	0	0	0	1 (11.1)	0	0	1 (12.5)	0	0	0
Stomatitis	0	0	0	0	0	0	0	0	1 (12.5)	0	0	0
Febrile neutropenia	0	0	0	0	0	0	0	0	0	0	1 (33.3)	0
Fatigue	0	0	0	0	0	0	0	0	0	0	1 (33.3)	0
*SAEs, n (%)*												
Total patients	0	1 (33.3)	0	0	0	0	1 (25.0)	0	2 (25.0)	0	3 (100.0)	1 (16.7)
Sepsis	0	0	0	0	0	0	0	0	0	0	1 (33.3)	0
Febrile neutropenia	0	0	0	0	0	0	0	0	0	0	2 (66.7)	0
Agranulocytosis	0	0	0	0	0	0	0	0	0	0	1 (33.3)	0
Dyspnoea	0	1 (33.3)	0	0	0	0	0	0	0	0	0	0
Cardiac arrest	0	0	0	0	0	0	0	0	1 (12.5)	0	0	0
Hypotension	0	0	0	0	0	0	1 (25.0)	0	0	0	1 (33.3)	0
Renal failure	0	0	0	0	0	0	0	0	0	0	0	1 (16.7)
Infusion-related reaction	0	0	0	0	0	0	0	0	1 (12.5)	0	0	0
*AEs leading to discontinuation of study treatment, n (%)*												
Total patients	0	0	0	0	0	0	0	0	1 (12.5)	0	0	0
Infusion-related reaction	0	0	0	0	0	0	0	0	1 (12.5)	0	0	0

*AE* adverse event, *DLT* dose-limiting toxicity, *G-CSF* granulocyte colony-stimulating factor, *SAE* serious adverse event.

### Safety

**Fig. 1:**
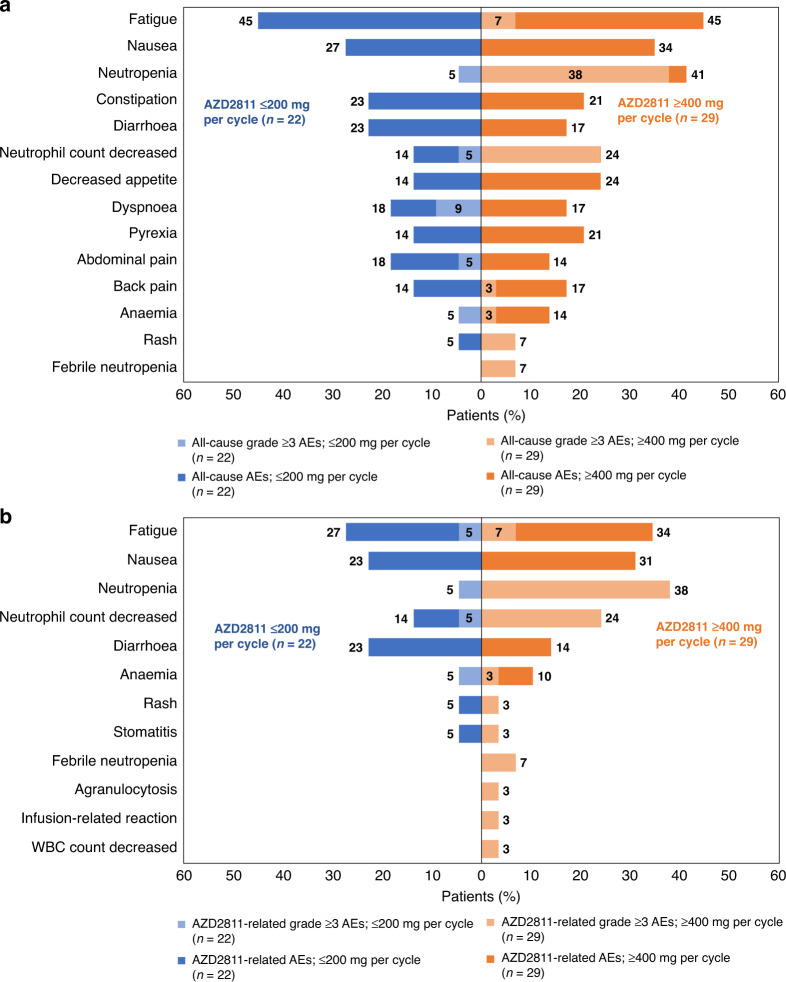
All-cause and treatment-related AEs. **a** All-cause AEs (all grades) occurring in ≥15% of patients and all-cause grade ≥3 AEs occurring in ≥2 patients, and **b** treatment-related AEs (investigator-assessed; all grades) occurring in ≥15% of patients and treatment-related grade ≥3 AEs occurring in ≥1 patient.

All-cause AEs (Fig. 1a) were reported in 100% of patients who received ≤200 mg AZD2811 per cycle (Cohorts 1–5 and 7–8; *n* = 22), and 96.6% of patients who received ≥400 mg AZD2811 per cycle (Cohorts 6 and 9–12; *n* = 29). The most common all-cause AEs in the ≤200 mg/cycle cohorts were fatigue (45.45%), nausea (27.3%), constipation (22.7%), and diarrhoea (22.7%), while the most common AEs in the ≥400 mg/cycle cohorts were fatigue (44.8%), neutropenia (41.4%), nausea (34.48%), decreased appetite (24.1%), and decreased neutrophil count (24.1%). Grade ≥3 all-cause AEs occurred in 27.3% of patients in the ≤200 mg/cycle cohorts and 72.4% in the ≥400 mg/cycle cohorts; the most common were dyspnoea (9.1%) in the former and neutropenia (37.9%) and decreased neutrophil count (24.1%) in the latter, indicating the need for G-CSF at higher doses.

AEs related to AZD2811 (Fig. 1b) occurred in 90.9% of patients in the ≤200 mg/cycle cohorts and 82.8% of patients in the ≥400 mg/cycle cohorts. The most common AZD2811-related AEs were fatigue (27.3%), nausea (22.7%), diarrhoea (22.7%), cough (13.6%), and decreased neutrophil count (13.6%) in the ≤200 mg/cycle cohorts, and neutropenia (37.9%), fatigue (34.48%), nausea (31.0%), decreased neutrophil count (24.1%), diarrhoea (13.8%), and decreased appetite (13.8%) in the ≥400 mg/cycle cohorts. Grade ≥3 AZD2811-related AEs occurred in 13.6% of patients in the ≤200 mg/cycle cohorts (anaemia, fatigue, neutropenia, and decreased neutrophil count in 1 patient [4.5%] each) and 65.5% of patients in the ≥400 mg/cycle cohorts (most commonly neutropenia [37.9%], decreased neutrophil count [24.1%], fatigue [6.9%], and febrile neutropenia [6.9%]).

Serious AEs (SAEs; Table 2) occurred in 2 patients (9.1%) in the ≤200 mg/cycle cohorts and 6 patients (20.7%) in the ≥400 mg/cycle cohorts. Neither of the SAEs in the ≤200 mg/cycle cohorts were related to AZD2811, but the SAEs in 3 (10.3%) patients in the ≥400 mg/cycle cohorts were related to AZD2811: febrile neutropenia and agranulocytosis in 1 patient in Cohort 11; febrile neutropenia alone in 1 patient in Cohort 11; and an infusion-related reaction in 1 patient in Cohort 9 (this was the only report of an infusion-related reaction in the study and led to treatment discontinuation). Two deaths due to AEs occurred on treatment but were not considered related to AZD2811: one was due to cardiac arrest (Cohort 9) and the other was due to sepsis (Cohort 11).

Dose interruptions due to AEs occurred in 3 patients (13.6%) receiving ≤200 mg/cycle: grade 2 rash (initially recorded as treatment-related, but later found to be not related) and grade 1 nasal congestion and oropharyngeal pain (not treatment-related) in 1 patient in Cohort 1; grade 1 increased blood creatinine and grade 2 AST elevation (not treatment-related) in 1 patient in Cohort 3; and grade 2 ALT and AST elevations (not treatment-related) in 1 patient in Cohort 4. Dose interruptions due to AEs occurred in 3 patients (10.3%) receiving ≥400 mg/cycle: grade 3 agranulocytosis and grade 3 febrile neutropenia (both treatment-related) in 1 patient in Cohort 11; grade 3 hypoxia (not treatment-related) in 1 patient in Cohort 6; and grade 3 pathological fracture (not treatment-related) in 1 patient in Cohort 11.

Dose reduction due to AZD2811-related AEs occurred in 1 patient (4.5%) in the ≤200 mg/cycle cohorts: grade 3 neutropenia in Cohort 8, requiring reduction from 200 to 100 mg in Cycle 3. Dose reductions due to AZD2811-related AEs occurred in 5 patients (17.2%) in the ≥400 mg/cycle cohorts: doses were reduced from 200 mg (on Days 1 and 4) to 100 mg in 3 patients in Cohort 6 due to grade 3 fatigue, grade 4 neutropenia, and grade 4 decreased neutrophil count (each *n* = 1) in Cycles 3, 2, and 2, respectively; from 400 to 200 mg in Cycle 2 due to grade 4 decreased neutrophil count in 1 patient in Cohort 9; and from 600 to 400 mg in Cycle 2 due to grade 4 neutropenia and grade 3 fatigue in 1 patient in Cohort 11. No patient required more than one dose reduction. One patient in Cohort 11 experienced febrile neutropenia in Cycle 1 but, due to a dosing error, continued to receive a dose of 600 mg in Cycle 2 instead of a reduced dose of 400 mg (according to the toxicity management guidance); this patient died due to sepsis, considered unrelated to AZD2811.

**Fig. 2:**
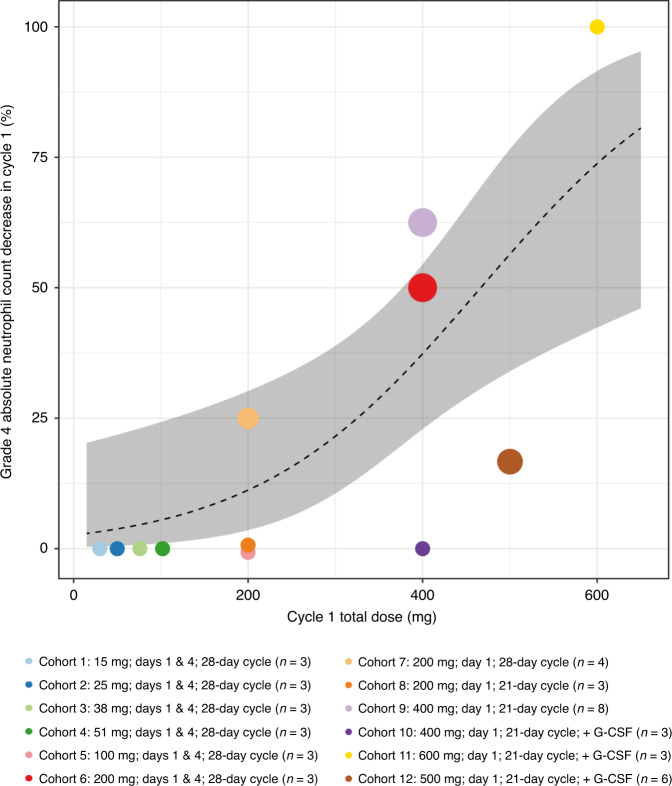
Dose–response for grade 4 decreases in absolute neutrophil count in Cycle 1. Based on ANC laboratory data. Figure shows the percentage grade 4 ANC decreases per cohort in Cycle 1 (coloured dots) versus planned total Cycle 1 dose. A grade 4 ANC decrease was defined as an ANC < 500 cells/μL post-baseline. The size of the coloured dots represents the relative cohort size. The black dashed line and grey shaded area represent the best fit of the data and 95% confidence interval based on simple logistic regression, respectively. Patients without ANC observations in Cycle 1 were excluded. ANC absolute neutrophil count, G-CSF granulocyte colony-stimulating factor.

Throughout dose escalation, absolute neutrophil count (ANC) was used to monitor bone marrow toxicity and as a marker for pharmacodynamic activity. Counts <500 cells/μL were observed at doses ≥200 mg/cycle and were more common at higher doses, with acceptable rates of grade 4 decreases in ANC at doses up to 500 mg/cycle (Fig. 2). Administration of G-CSF on Day 8 supported ANC recovery before subsequent cycles (Supplementary Fig. 2A–C), allowing tolerability of the RP2D of 500 mg on Day 1 of each 21-day cycle.

### PK parameters

**Fig. 3:**
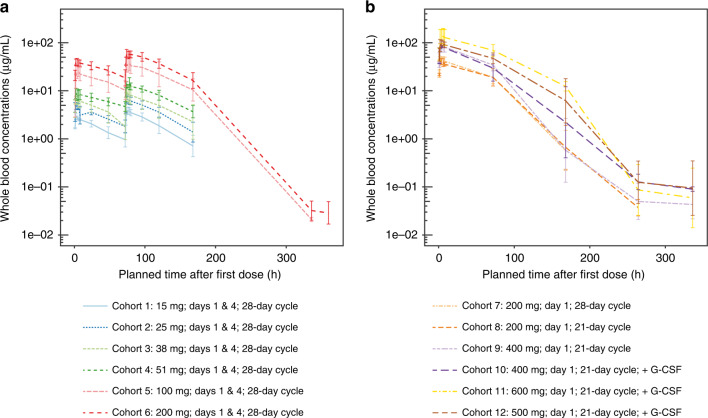
Geometric mean (±geoSD) AZD2811 whole blood concentrations. **a** For days 1 and 4 regimens and **b** for day 1 only regimens. In Cohorts 1–6, PK blood samples (4 mL per sample) for determination of AZD2811 blood concentrations were collected from all patients on Cycle 1 Day 1 pre-dose, and at 1, 2, 4, 6, 8, 24, and 48 h (±15 min for each time point) from the start of infusion, then on Cycle 1 Day 4 pre-dose, and at 1, 2, 4, 6, 8, and 24 h (day 5), 48 h (day 6), 96 h (day 8) from the start of infusion, and in the morning on days 15, 16, and 22. A Cycle 2 Day 1 pre-dose sample and a 2-h sample (end of infusion) were also obtained. In Cohorts 7–12, PK blood samples were collected from all patients on Cycle 1 Day 1 pre-dose, and at 1, 2, 4, 6, and 8 h from the start of infusion, and in the morning on Days 4, 8, 12, and 22. G-CSF granulocyte colony stimulating factor, PK pharmacokinetics, Q3W every 3 weeks, Q4W every 4 weeks.

Compartmental PK analysis was used to derive AUC from time zero to the time of the last planned sampling time in the study (AUC_0–504h_). *C*_max_ and *t*_max_ were derived using the standard non-compartmental approach. PK parameters of *C*_max_, *t*_max_, and AUC_0–504h_ are summarised by cohort in Supplementary Table 2, and geometric mean AZD2811 whole blood concentrations are presented by cohort in Fig. 3. Maximum AZD2811 whole blood concentrations were observed after the end of infusions (Days 1 and 4). Following a 2-h infusion on Day 1, AZD2811 blood exposure was sustained for several days, with a gradual and monophasic decline in concentration post-infusion until approximately 96 h. There was evidence of a second and third phase, 96 and 264 h post-dose. AZD2811 PK appeared broadly dose-proportional across the dose range evaluated (Supplementary Fig. 3). Urine samples were only collected in the first four cohorts, and AZD2811 concentrations were below the limit of quantification in the majority (>80%). Consequently, only the total amount of unchanged AZD2811 excreted into urine over 0–72 h was calculated; the maximum amount excreted was 294.40 µg for a patient in Cohort 4 (51 mg AZD2811), which represented <1% of the dose.

### Preliminary antitumour activity

Best overall response is shown in Table 3 and duration of treatment is shown in Supplementary Fig. 4. One patient with prostate cancer in Cohort 6 had a confirmed PR; there were no CRs. The rate of SD (≥8 weeks for patients treated on a 28-day cycle, ≥6 weeks for patients treated on a 21-day cycle) across all cohorts was 45.1%. Most patients with sustained SD received ≥100 mg/cycle.

**Table 3:** Best overall response (evaluable-for-efficacy analysis set).

	Total (*N* = 51) *n* (%)
Partial response	1 (2.0)
Stable disease ≥2 cycles	23 (45.1)
Progressive disease	22 (43.1)
RECIST progression	19 (37.3)
Death	3 (5.9)
Not evaluable	5 (9.8)
Stable disease <2 cycles	1 (2.0)
Incomplete post-baseline assessments	4 (7.8)

Two cycles defined as 56 days in 28-day cycles; 42 days in 21-day cycles.

Notably, the patient in Cohort 6 with a PR had an 80% reduction from baseline in sum of target lesion diameters. The first two scans showed SD, then a PR was observed at the third scan. He remained on treatment for a further 40 weeks and then discontinued. He had previously received 3 treatment regimens for metastatic prostate cancer (bicalutamide, leuprolide acetate, and abiraterone acetate), and had a best overall response of SD prior to progression on all 3 regimens with a rapid rise of prostate-specific antigen reported with the bicalutamide regimen. A tissue sample from this patient showed three distinct AR mutations, a BRCA2-ERG rearrangement and a TMPRSS2 rearrangement; plasma samples showed mutations in ARID1A, MET, RB1, AR and CDKN2A. Germline testing showed a BRCA2 deletion/duplication. Additionally of note, 1 patient with cancer of pancreaticobiliary origin in Cohort 8 had SD for 66 weeks; this patient had a best overall response of PR or non-CR/non-PD before progression on 3 prior treatment regimens (FOLFIRINOX [folinic acid, fluorouracil, irinotecan, and oxaliplatin], capecitabine, and paclitaxel plus gemcitabine followed by gemcitabine). Another patient with adenocarcinoma of the breast in Cohort 7 had the longest duration of therapy of >17 months, and a best overall response of SD. The patient had oestrogen receptor-positive, progesterone receptor-positive disease, and HER2 non-amplified status by dual in situ hybridisation. Immunohistochemistry staining showed the tumour was positive for AE1/3, CK7, GATA-3 and GCDFP-15; and was negative for CK20, PAX-8, CDX-2, WT-1, TTF-1, mammaglobin and BRAF V600E. Genomic profiling showed a TP53 R723H mutation.

## Discussion

In the dose-escalation phase of this first-in-human study, AZD2811 was assessed in 12 cohorts; the MTD and RP2D were determined to be 500 mg on Day 1 of a 21-day cycle with mandatory combination G-CSF. Administration of AZD2811 only on Day 1 of each cycle (rather than on Days 1 and 4) was implemented alongside the addition of G-CSF prophylaxis with the aim of managing recovery from neutropenia and improving patient convenience, and may also facilitate potential combinations with other treatments in future studies. A cycle length of 21 days rather than 28 days was deemed to be preferable to minimise the fall in drug concentrations below the active level between cycles; this increase in dose intensity was considered particularly important for the treatment of patients with rapidly growing tumours such as SCLC, ovarian cancer, and aggressive lymphomas. Mandatory G-CSF (administered on Day 8 of each cycle) minimised the impact of treatment-related neutropenia/decreased neutrophil count at higher doses. The MTD/RP2D was investigated in the expansion phase of this study (to be published separately) in patients with previously treated SCLC (both limited-stage [LS] and extensive-stage [ES]), whose tumours are expected to be sensitive to AURKB inhibition due to the characteristic rapid proliferation and reliance on accelerated mitosis of SCLC. The dosing schedule assessed in Cohort 6 (200 mg AZD2811 on Days 1 and 4 every 28 days) was also explored in the N3 cohort of the SCLC Umbrella Korea Studies (SUKSES-N3), a phase 2 trial of second- or third-line therapy for LS- or ES-SCLC. There were no objective responses but 5/15 (33.3%) patients had SD [25]. Other dosing schedules are also being explored in a phase 1/2 study (NCT03217838) of AZD2811 as monotherapy or in combination with azacitidine or venetoclax in patients with AML or myelodysplastic syndrome (MDS); initial safety data have been published [26].

In previous phase 1 studies in patients with advanced solid tumours, AURKB inhibitors have been generally well tolerated, with bone marrow and gastrointestinal toxicities typically being the most common AEs [15, 18, 19, 21]. AZD2811 monotherapy was tolerable with G-CSF support in the current study, and AEs were similar in type and frequency to the previous phase 1 studies. In contrast to chemotherapy, there was little evidence of cumulative toxicity with AZD2811; dose modifications (interruptions or reductions) were rare and several patients remained on treatment for long periods, including 3 who stayed on treatment for over 1 year. Unlike previous experience with barasertib [17], stomatitis was not a prominent AE. In SUKSES-N3, 9/15 (60.0%) patients without prophylactic G-CSF had target-related grade ≥3 neutropenia, of whom 6 (40.0%) had febrile neutropenia, including 1 (6.7%) with septic shock due to pneumonia with grade 4 neutropenia [25]. In patients receiving ≥400 mg/cycle in the current study, grade ≥3 AZD2811-related neutropenia occurred in 37.9%, decreased neutrophil count in 24.1%; and febrile neutropenia in 6.9% of patients. One patient with febrile neutropenia died due to sepsis, which was considered unrelated to AZD2811.

Based on the key role of AURKB in mitosis, neutropenia and decreased neutrophil count are expected toxicities of treatment with AURKB inhibitors due to the rapid rate of cell division in bone marrow neutrophils [2]. On-target neutrophil count decrease or neutropenia were therefore used as a surrogate pharmacodynamic marker. All patients who experienced clinical benefit and extended treatment durations received AZD2811 doses associated with neutropenia. Neutropenia was not observed in Cohorts 1–5, but emerged at high rates in Cohort 6 (55.6%, all grade 3/4), and Cohorts 7–12 (29.6%, nearly all grade 3/4). These results imply that AZD2811 doses >200 mg/cycle elicit a pharmacological response in bone marrow. To mitigate the effects of neutropenia, G-CSF was added to the treatment regimens in several cohorts, and was shown to support ANC recovery before subsequent cycles, preventing cumulative toxicity. Neither the grade nor the depth of ANC reduction were related to the number of cycles, indicating the absence of cumulative toxicity. Dose reductions due to AEs were reported only in patients receiving AZD2811 doses ≥200 mg/cycle.

Other AURKB inhibitors have demonstrated limited antitumour activity in phase 1 studies in patients with advanced solid tumours. For example, with BI 831266, 1 patient (4%) experienced a PR and 4 patients (16%) had SD [19], while with BI 811283, no objective responses were reported but 30–33% of patients had SD, depending on the treatment schedule [21]. Similarly, in the phase 1 study of barasertib in advanced solid tumours, there were no objective responses, and SD was reported in 23% of patients [18]. However, the efficacy of barasertib was considerably more promising in patients with AML [17], prompting the development of the nanoparticle-encapsulated form of AZD2811, with the aim of increasing biodistribution to tumours and maximising the therapeutic effect while reducing toxicity by sparing healthy tissue [23]. In our study, preliminary antitumour activity was greater than that observed in previous studies, with 1 patient having a PR and nearly half (45.1%) of patients having SD. This observation aligns with preclinical results in which the nanoparticle formulation of AZD2811 displayed slower onset and more prolonged inhibition of AURKB in tumours compared with barasertib, together with more effective tumour growth inhibition in multiple models [23]. It is not immediately clear whether the enhanced antitumour activity of AZD2811 compared with barasertib is entirely attributable to the nanoparticle formulation, or whether the higher doses accessible in the presence of G-CSF prophylaxis are also impacting efficacy. This was explored further in the dose-expansion part of this study in patients with SCLC (to be published separately), which assessed the pharmacodynamic effects of AURKB inhibition to assess AZD2811 target inhibition and its downstream effect in tumour tissue. Further insight may also be gained from the assessment of nanoparticle-encapsulated AZD2811 in patients with AML or MDS [26].

With respect to the patient with PR, we hypothesise that the RB1 mutation or the BRCA2 deletion/duplication may be the most relevant alterations associated with this patient’s response, as synthetic lethality between RB1 deficiency and Aurora B kinase inhibitors [27], as well as between BRCA1/2 mutations and DDR pathway inhibitors, has been reported previously [28, 29].

Analyses of PK data demonstrated that maximum AZD2811 whole blood concentrations were observed after drug infusion. The long-circulating nanoparticle formulation provided more sustained and extended total AZD2811 blood exposure compared with historical measurements in barasertib clinical studies [15, 16, 30]. As such, the sustained exposure of the nanoparticle formulation may address some of the issues observed with the barasertib formulation, including long infusion times required for target coverage and low therapeutic index.

This study has some limitations. This efficacy analysis was limited by the small and heterogeneous population that prevented reliable identification of subgroups of patients who may respond to AZD2811. At the time of study initiation, there were no predictive biomarkers for AURKB inhibitors, but research is underway to identify potential biomarkers and molecularly defined patient subsets who may be sensitive to AZD2811 [31, 32]. While AZD2811 antitumour activity appears limited, it is possible that enhanced efficacy could be achieved at higher doses; however, it is not feasible to assess this due to dose-limiting neutropenia, even in the presence of G-CSF prophylaxis. The strength of AZD2811 may be its ability to provide durable tumour control, as seen in almost half of patients in our study.

In conclusion, 500 mg of AZD2811 in a 21-day cycle dosed on Day 1, with G-CSF support on Day 8, was tolerable in patients with advanced solid tumours. On-target neutropenia was a pharmacodynamic biomarker and was manageable with the addition of G-CSF. This RP2D has been investigated in the dose-expansion phase of the study in patients with SCLC and will be published separately (Johnson et al., manuscript in preparation).

## Supplementary information

Supplementary Figure 1

Supplementary Figure 2

Supplementary Figure 3

Supplementary Figure 4

Supplementary Materials

## Data Availability

Data underlying the findings described in this manuscript may be obtained in accordance with AstraZeneca’s data sharing policy described at https://astrazenecagrouptrials.pharmacm.com/ST/Submission/Disclosure.
