# Supplementary figures and images for: Safety, tolerability, and pharmacokinetics of Aurora kinase B inhibitor AZD2811: a phase 1 dose-finding study in patients with advanced solid tumours
Source: Br J Cancer. 2023 Mar 4;128(10):1906–15. doi: 10.1038/s41416-023-02185-2 (PMC10147685; doi:10.1038/s41416-023-02185-2)

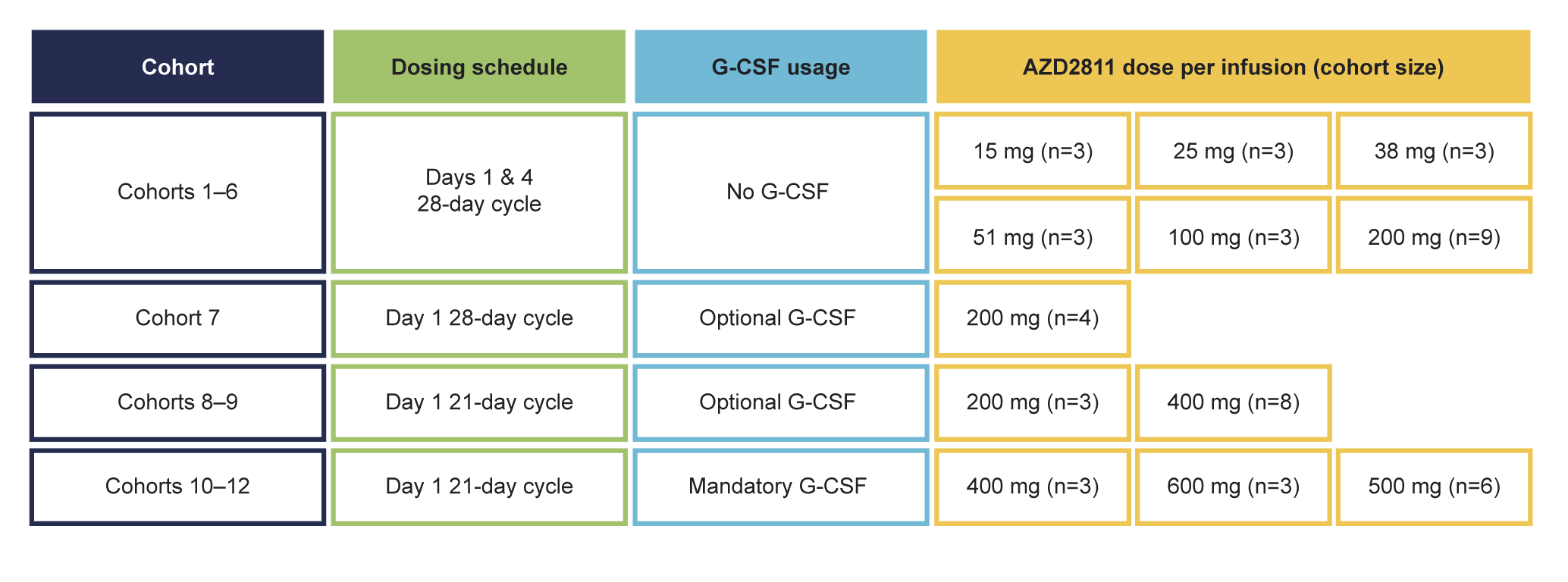

Supplement: Supplementary file 1 — Supplementary Figure 1 [file 41416_2023_2185_MOESM1_ESM.tif]

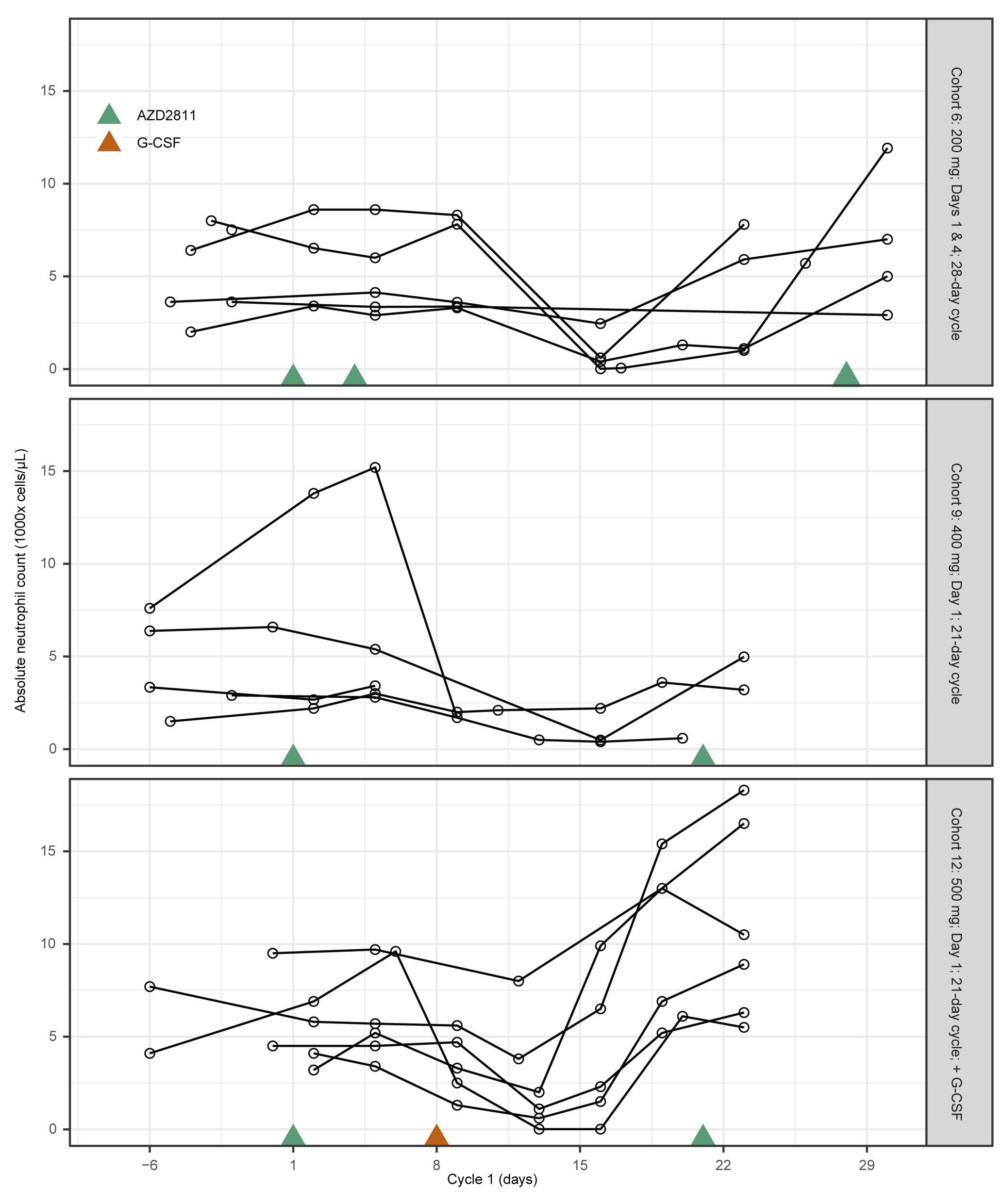

Supplement: Supplementary file 2 — Supplementary Figure 2 [file 41416_2023_2185_MOESM2_ESM.tif]

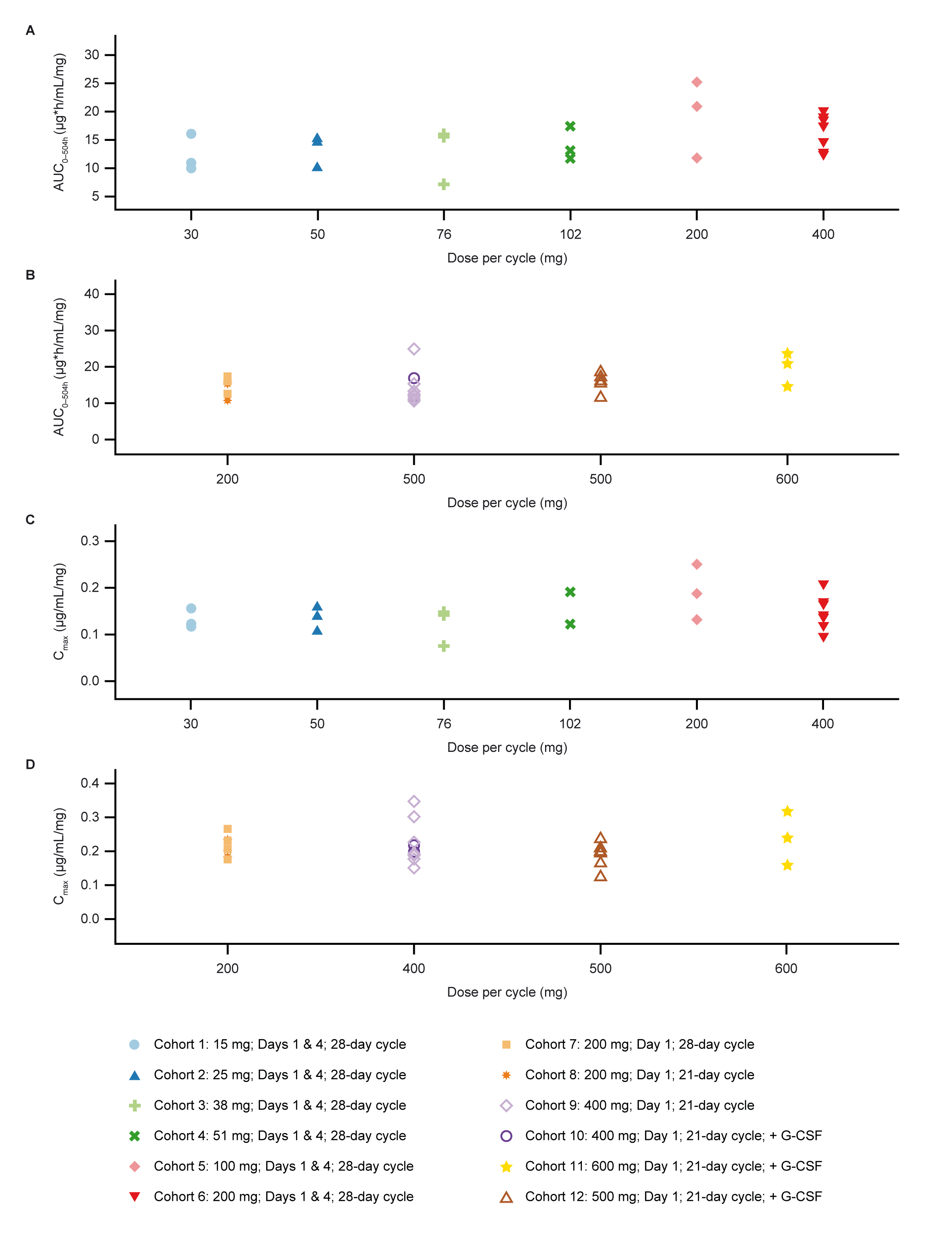

Supplement: Supplementary file 3 — Supplementary Figure 3 [file 41416_2023_2185_MOESM3_ESM.tif]

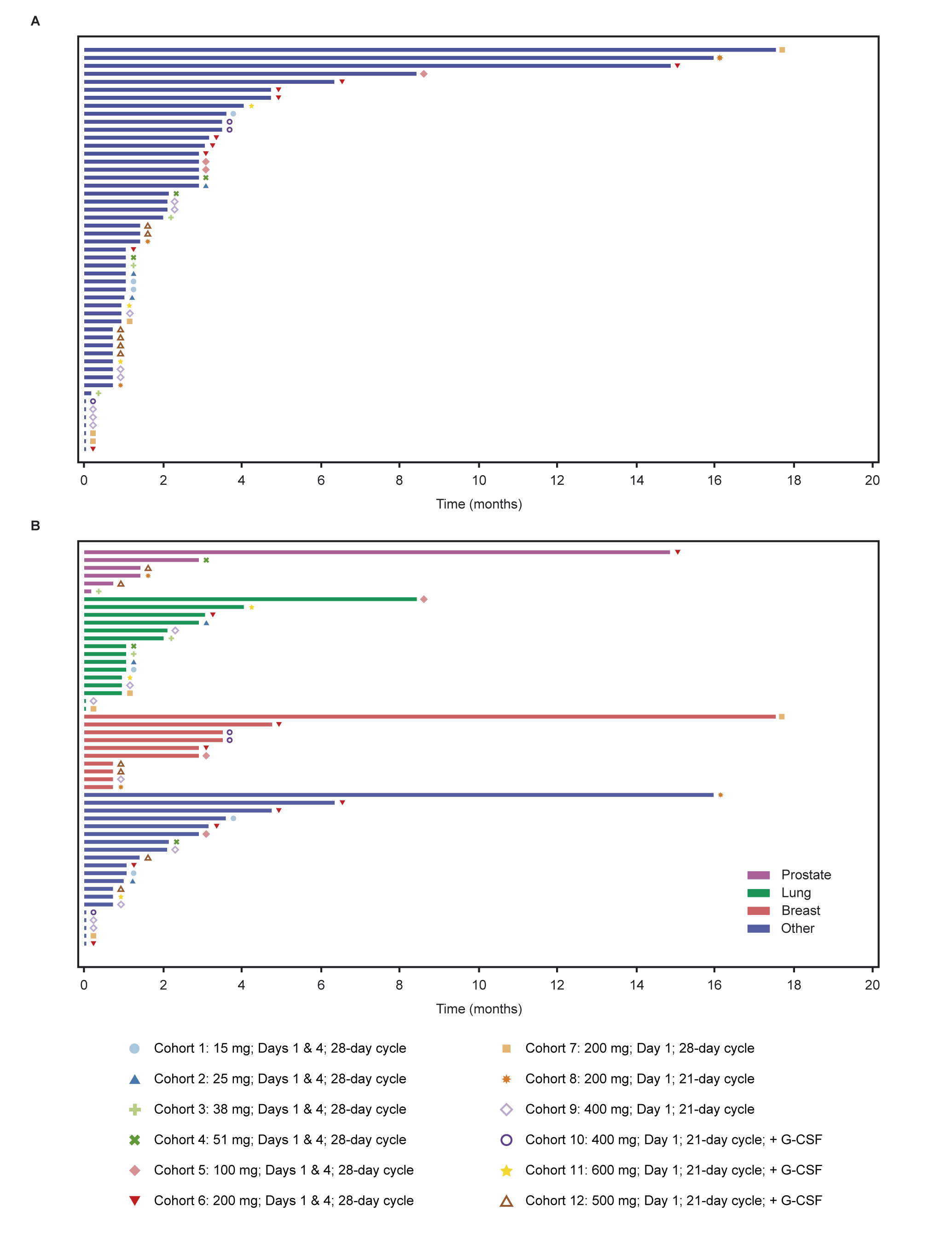

Supplement: Supplementary file 4 — Supplementary Figure 4 [file 41416_2023_2185_MOESM4_ESM.tif]
