# Supplementary material for: Safety, tolerability, and pharmacokinetics of Aurora kinase B inhibitor AZD2811: a phase 1 dose-finding study in patients with advanced solid tumours
Source: Br J Cancer. 2023 Mar 4;128(10):1906–15. doi: 10.1038/s41416-023-02185-2 (PMC10147685; doi:10.1038/s41416-023-02185-2)
Supplement: Supplementary file 5 — Supplementary Materials [file 41416_2023_2185_MOESM5_ESM.docx]

**SUPPLEMENTARY MATERIAL**

**Supplementary Table 1**. Summary of dose-limiting toxicity events.

| Cohort | AZD2811 dose | Patients  enrolled | Evaluable  patients | Evaluable  patients  with DLTs | Details of DLTs |
| --- | --- | --- | --- | --- | --- |
| Cohort 1 | 15 mg (Days 1 & 4, 28-day cycles) | 3 | 3 | 0 |  |
| Cohort 2 | 25 mg (Days 1 & 4, 28-day cycles) | 3 | 3 | 0 |  |
| Cohort 3 | 38 mg (Days 1 & 4, 28-day cycles) | 3 | 3 | 0 |  |
| Cohort 4 | 51 mg (Days 1 & 4, 28-day cycles) | 3 | 3 | 0 |  |
| Cohort 5 | 100 mg (Days 1 & 4, 28-day cycles) | 3 | 3 | 0 |  |
| Cohort 6 | 200 mg (Days 1 & 4, 28-day cycles) | 9 | 6 | 1 | Grade 4 decreased neutrophil count, duration 12 days (*n* = 1) |
| Cohort 7 | 200 mg (Day 1, 28-day cycles; optional G-CSF) | 4 | 3 | 0 |  |
| Cohort 8 | 200 mg (Day 1, 21-day cycles; optional G-CSF) | 3 | 3 | 0 |  |
| Cohort 9 | 400 mg (Day 1, 21-day cycles; optional G-CSF) | 8 | 6 | 2 | Grade 3 stomatitis (*n* = 1) Grade 4 decreased neutrophil count, duration 12 days (*n* = 1) |
| Cohort 10 | 400 mg (Day 1, 21-day cycles; mandatory G-CSF) | 3 | 3 | 0 |  |
| Cohort 11 | 600 mg (Day 1, 21-day cycles; mandatory G-CSF) | 3 | 3 | 2 | Grade 3 febrile neutropenia (*n* = 1)  Grade 3 fatigue (*n* = 1) |
| Cohort 12 | 500 mg (Day 1, 21-day cycles; mandatory G-CSF) | 6 | 6 | 0 |  |

*DLT* dose-limiting toxicities, *G-CSF* granulocyte colony-stimulating factor

**Supplementary Table 2**. PK parameters by cohort.

|  | **Days 1 and 4, 28-day cycles** | | | | | | **Day 1,**  **28-day cycles** | **Day 1, 21-day cycles** | | | | |  |
| --- | --- | --- | --- | --- | --- | --- | --- | --- | --- | --- | --- | --- | --- |
|  | **No G-CSF** | | | | | | **Optional G-CSF** | | | **Mandatory G-CSF** | | |  |
| **PK parameter** | **Cohort 1: AZD2811**  **(15 mg)**  **(*N* = 3)** | **Cohort 2: AZD2811**  **(25 mg)**  **(*N* = 3)** | **Cohort 3: AZD2811**  **(38 mg)**  **(*N* = 3)** | **Cohort 4: AZD2811**  **(51 mg)**  **(*N* = 3)** | **Cohort 5: AZD2811**  **(100 mg)  (*N* = 3)** | **Cohort 6: AZD2811**  **(200 mg)  (*N* = 9)** | **Cohort 7: AZD2811**  **(200 mg)  (*N* = 4)** | **Cohort 8: AZD2811**  **(200 mg)**  **(*N* = 3)** | **Cohort 9: AZD2811**  **(400 mg)  (*N* = 7)** | **Cohort 10: AZD2811**  **(400 mg)  (*N* = 2)** | **Cohort 11: AZD2811**  **(600 mg)  (*N* = 3)** | **Cohort 12: AZD2811**  **(500 mg)  (*N* = 6)** | |
| Geometric mean C_max_, µg/mL (CV%) | 3.9 (15.8) | 6.7 (19.7) | 8.9 (38.4) | 14.5 (25.3) | 36.8 (33.4) | 58.3 (22.9) | 43.4 (17.6) | 41.4 (11.9) | 87.3 (30.4) | NC | 138.2 (35.7) | 93.9 (22.0) | |
| Median t_max_, h (range) | 77 (75–77) | 75 (75–76) | 75 (75–99) | 78 (75–78) | 78 (78–78) | 74 (8–79) | 2 (2–2) | 4 (2–6) | 3 (2–6) | NC | 6 (4–8) | 6 (2–97) | |
| Geometric mean AUC_(0–504h)_, µg*h/mL (CV%) | 362.2 (25.1) | 658.0 (22.9) | 917.0 (48.6) | 1416 (20.3) | 3671 (41.1) | 6225 (20.8)^a^ | 3020 (16.8)^b^ | 2753 (22.2) | 5445 (29.8) | NC | 11600 (25.4) | 7845 (18.1)^c^ | |

*AUC* area under the curve, *C_max_* maximum concentration, *CV* coefficient of variation, *NC* not calculable as *N* < 3, *PK* pharmacokinetic, *t_max_* time to maximum concentration.

^a^*N* = 8, ^b^*N* = 3, ^c^*N* = 5.

**Supplementary Fig. 1.** Study design, dose-escalation phase.

*G-CSF* granulocyte colony-stimulating factor.

**Supplementary Fig. 2.** Patient-level neutrophil profiles in Cycle 1 from (**A**) Cohort 6, (**B**) Cohort 9, and (**C**) Cohort 12.

Figure shows patient-level neutrophil profiles in Cycle 1 from Cohort 6 (**Panel A**), Cohort 9 (**Panel B**), and Cohort 12 (**Panel C**). The coloured triangles represent the timings of AZD2811 and G-CSF dosing. For comparison of cohorts with and without G-CSF, 3 patients from Cohort 6 and 3 patients from Cohort 9 receiving G-CSF during Cycle 1 were excluded.

*G-CSF* granulocyte colony-stimulating factor; *Q3W* every 3 weeks, *Q4W* every 4 weeks.

**Supplementary Fig. 3.** Dose-normalised PK parameters versus dose for (**A**) AUC_0–504h_ in Cohorts
1–6, (**B**) AUC_0–504h_ in Cohorts 7–12, (**C**) C_max_ in Cohorts 1–6, and (**D**) C_max_ in Cohorts 7–12.

*G-CSF* granulocyte colony-stimulating factor, *PK* pharmacokinetics.

**Supplementary Fig. 4.** Swimmer plot showing duration of therapy in (**A**) all treated patients and (**B**) by primary diagnosis group.
